# Experimental Characterisation of Lime-Based Textile-Reinforced Mortar Systems Made of Either Jute or Flax Fabrics

**DOI:** 10.3390/ma16020709

**Published:** 2023-01-11

**Authors:** Marco Pepe, Rosario Lombardi, Giuseppe Ferrara, Stefano Agnetti, Enzo Martinelli

**Affiliations:** 1 DICiv—Department of Civil Engineering, University of Salerno, Via Giovanni Paolo II, 132, 84084 Fisciano, Italy; 2 TESIS srl, Via Giovanni Paolo II, 132, 84084 Fisciano, Italy; 3 DISAT—Department of Applied Science and Technology, Politecnico di Torino, Corso Duca Degli Abruzzi, 24, 10129 Torino, Italy; 4 Kimia s.p.a., Via del Rame, 73, 06134 Perugia, Italy

**Keywords:** TRM, composites, strengthening, natural fibres, sustainability

## Abstract

Existing buildings are often in need of strengthening interventions, and several technical solutions have been recently developed for this purpose. Among them, the use of textile-reinforced mortar (TRM) composites has gained consensus as a technically viable and economically convenient option. Moreover, TRM has the potential to be employed as a reversible and sustainable strengthening technique for masonry buildings. In this context, the present paper aims to investigate the mechanical properties of TRM systems consisting of sustainable phases, such as lime-based matrices and natural fabrics produced by waiving fibers obtained from plants, such as Jute or Flax. This class composite system can be referred to as natural TRM and is denoted by the acronym NTRM. The present study moves from the geometric and mechanical characterisation of fibres and fabrics and, after having also investigated the properties of the mortar, it reports the results of tensile tests carried out on specimens of the NTRM systems under consideration, with the main aim of providing the empirical bases of the relationships between the geometric and physical properties of the constituents and the resulting mechanical response of the composite system. The obtained results show that the considered Flax-TRM system has an apparent composite behavior, as its response to tension is clearly characterised by the well-known three stages corresponding to the elastic response, the formation of cracks, and the reinforcement response up to rupture. Conversely, the Jute-TRM system needs to be further improved in terms of balance between the properties of the matrix and the internal reinforcement. Further studies will be devoted to this specific aspect and, more generally, to investigating the relationships between constituents’ properties and the NTRM behavior.

## 1. Introduction

The recent seismic events that have occurred in Italy (Aquila in 2009, Emilia-Romagna 2012, Central Italy 2016.) have widely demonstrated how existing masonry buildings, yet generally characterised by sufficient bearing capacities under gravitational loads, are often deficient in terms of seismic response. This deficiency emerges quite regularly as post-earthquake damage is observed, which often highlights both in-plane and out-of-plane failure mechanisms in the main walls of masonry buildings [1].

Therefore, masonry structures are widely in need of strengthening in terms of seismic capacity, and hence, several technical solutions are currently available to practitioners for that purpose. Among those solutions, the use of textile-reinforced mortar (TRM) systems have emerged as a technically viable and economically convenient opinion for seismic strengthening [2]. TRM systems consist of two “phases”:

- An inorganic matrix, which is generally represented by a cement- or lime-based mortar, the use of hydraulic lime being more and more common [3,4,5,6];
- An internal reinforcement consisting of a low-density textile made of various types of fibres, such as glass, basalt, carbon, high-strength steel, PBO (p-Phenylene Benzobis Oxazole), and aramid [1,7].

Several reasons have driven the diffusion and growing consensus of TRM systems as a strengthening technique for masonry structures. Among other things, TRM systems can be easily put in place, as their application basically requires the same skills needed for workers to realise plaster layers. Therefore, they lead to inexpensive and reversible strengthening interventions [2]. Furthermore, with reference to masonry, the use of lime-based mortars brings further advantages, such as higher compatibility between TRM and the supporting material, higher permeability, and better fire resistance with respect to the initially more common fibre-reinforced polymer (FRP) composites [1].

TRM systems are mainly employed to strengthen masonry walls, arches, and vaults; they are also used to confine masonry columns [7,8,9].

Therefore, given their diffusion over the years, it has proven necessary to develop tests capable of characterising the mechanical response of the TRM systems, both in terms of their tensile behavior and with respect to the quality of the bond that is established between the composite material and the masonry itself (shear-bond test). Moreover, durability turns out to be one of the issues of main concern for TRM systems [1,2]. In this context, therefore, various standards have been developed, which, on the one hand, define the criteria for the qualification of TRMs, and, on the other hand, provide practitioners with indications regarding their design and application [1,3,7,9,10,11,12,13,14,15,16].

Having said that, in recent years, the concept of sustainability has emerged as a paradigm for all human activities, and the construction sector is striving to meet this new challenge. This led researchers to focus on interventions that could be increasingly compatible with the aforementioned objectives [2]. With specific reference to the masonry, in particular, attention was paid to the study of TRMs that were always made up of an inorganic matrix, while the internal reinforcement was replaced by fabrics of vegetal origisn, such as jute, flax, sisal, coir, hemp, and curauá [2]. TRM, including these kinds of fabrics, is referred to as natural TRM (NTRM) [1,2,3].

Additionally, in this case, the studies carried out so far mainly deal with determining the relevant mechanical properties of the TRM systems, especially in terms of tensile and bending behaviour. To this aim, many different plant fibres were considered, such as jute, sisal, coir, flax, hemp, and curaua [17,18,19,20]. Tensile tests on NTRM, although showing a capability to fully exploit the fabric strength, also emphasised some discrepancies in the mechanical behaviour with respect to the more traditional composites and internally reinforced synthetic fibres. In fact, NTRM systems tend to exhibit larger displacements and more significant drops in the load in correspondence to crack onset [21]. Moreover, it was emphasised that the fabric volume fraction plays a key role in the overall mechanical response of the composite. Thus, reinforcement amount and matrix thickness need to be properly defined to produce a mechanically efficient system [2,17,21]. Impregnation treatments of natural fabrics are a promising solution to improve the mechanical behaviour, as they lead to an increase in the stiffness of the textile with the consequent reduction in the crack opening width and in the overall displacements of the composite [2,3]. However, issues related to the fibre–to–matrix bond also arose due to coating treatments [22].

Furthermore, the bond between the fabric and matrix was investigated by carrying out tests on its unthreading and the adhesion of the NTRMs and the masonry [2].

The obtained results have shown that these solutions have excellent resistance, which is comparable to the behavior of conventional TRMs. Conversely, critical issues have emerged, especially related to the variability of the natural fibre properties, which has led to a higher inhomogeneity in the behavior of NTRMs compared to conventional TRMs [1].

Aiming at exploring the suitability of NTRM as a strengthening system for structural applications, many studies have been carried out to investigate the in-plane shear capacity of masonry elements when externally strengthened by plant-based composites showing an increase in their capacity due to the strengthening system [23,24]. Additionally, the investigation of the mechanical behavior of externally strengthened NTRM masonry elements subjected to eccentric loads resulted in an increase in ductility with respect to reference samples reinforced with synthetic fibre-based composites [25]. Masonry walls, reinforced by Flax-TRM composites, and subjected to cyclic loading conditions, exhibited a significant increase in the shear capacity and ultimate drift and promoted the development of energy dissipation mechanisms while ensuring structural integrity [26].

However, durability is a fundamental issue that emerged from studies concerning NTRMs [1,2,3]. In particular, it has been observed that these are extremely sensitive to alkaline environments, which can particularly damage the natural fabric inside the matrix [2]. In this sense, coating the fabric with polymers (e.g., epoxy resins) is a possible solution to protect it and, at the same time, increase its stiffness, which would lead to smaller deformations and lower load losses when the various cracks form during the traction of the NTRM [1,2,3].

The present study aims to further deepen the study of the mechanical characteristics of NTRM, specifically consisting of two alternative compositions for the inorganic matrix, consisting of a hydraulic lime-based mortar and an internal reinforcement made of either flax or jute fabrics. Specifically, *Limepor MT* and *Limepor EDO* mortars, produced by Kimia S.p.A. [5,6], were consistently considered as matrices for jute and flax reinforcements, respectively.

A full geometric and mechanical characterisation of the single constituents is proposed before investigating the post-cracking response of the NTRM specimens tested in tension. In addition to the analysis of the results, a comparison will be made between what has been obtained and the mechanical characteristics reported in the literature, referring both to other NTRMs and to conventional TRMs. Therefore, the main novelty of the present paper (besides all the experimental results which are new per se) is the comparative analysis of the structural behavior of a composite system and how this is intrinsically influenced by the geometric and mechanical properties of both the mortar matrix and the internal reinforcement fabric made of either flax or jute. Consequently, it sheds a critical light on the use of certain fabrics and points out the superiority of another one.

## 2. Materials and Methods

The present section describes the test methods adopted for the mechanical and physical characteristics of both the employed raw materials and the resulting TRM system.

### 2.1. Geometric Characterisation of Natural Yarns

**Figure 1:**
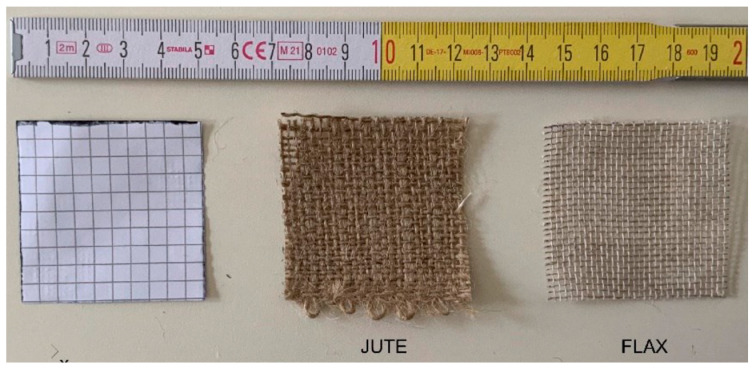
Fabric mesh.

The Jute and Flax fabrics under consideration in this paper are provided by Kimia s.p.a. (Perugia, Italy) as reinforcement textiles for inorganic matrix-based composites (Figure 1).

First of all, a geometric characterisation of the textile structure was performed. The textiles consist of bi-directional woven jute and flax fabric with a plain weave. Warp and weft yarns were arranged so that they realised a simple cross pattern. Two representative samples (50 mm × 50 mm) were considered (Figure 1), and, for each of them, the yarns in both directions were counted in order to define the mesh density.

Table 1 reports the main results of this analysis, which highlight how the flax textile mesh was denser than the jute one. In addition, it is worth mentioning that the jute yarn is extremely uneven and “frayed”, while the flax yarn is homogeneous and compact.

### 2.2. Physical Characterisation of Natural Yarns

In the following paragraph, the various tests performed to determine the physical characteristics of the fabrics are presented.

#### 2.2.1. Linear Density

The linear density was evaluated in accordance with EN ISO 1889: 2009 [27]. The basic principle of the test is to proceed with weighing a fabric sample of a known length and then determining the linear density. A total of five specimens were taken out of the textile, and the linear density *T_t_*, expressed in g/km, were calculated as follows:

(1) $$T_{t}=\frac{1000\times m}{L}$$

where:

- *m* is the mass, in gram, of the specimen;
- *L* is the length, in meters, of the specimen.

#### 2.2.2. Density

The ASTM D8171-18 standard (Archimedes Method) and the balance guidelines (“Sartorius YDK 01, YDK 01-0D, YDK 01LP-Density Determination Kit User’s Manual”) were used for the evaluation of the natural textile density [28,29].

The method proposed by the ASTM D8171-18 used Archimedes’ principle to measure the buoyancy generated by the saturated yarn immersed in a fluid, from which it is then possible to calculate the volume of the sample and, therefore, its density. It must be specified that the certified fluid indicated in the ASTM D8171-18 standard is soybean oil, while in this study, distilled water was used: this was performed in accordance with the manual of the machinery used [28].

The reference relation is shown below [29]:

(2) $$\rho =\frac{W\left(a\right)\times \left[\rho \left(fl\right)-\rho \left(a\right)\right]}{0.99983\times \left[W\left(a\right)-W\left(fl\right)\right]}$$

where:

- *ρ* is the solid specimen density;
- *ρ(fl)* is the fluid density;
- *ρ(a) =* 0.0012 g/cm^3^ is the air density measured at 20 °C and 101.325 kPa;
- *W(a)* is the weight of the solid specimen, in air;
- *W(fl)* is the weight of the solid specimen, in fluid;
- 0.99983 is the geometrical correction factor.

#### 2.2.3. Other Properties

The test results reported in Section 2.2.1 and Section 2.2.2 were also employed in calculating other properties, such as the density and linear density of the yarn in dry conditions, the cross-section of the filaments, and the absorption of fluid by the fabric.

1. Linear Density in dry conditions
  (3) $$TEX=\frac{W_{dry}}{L}$$
  where:
  - *TEX* is the linear density of the specimen in dry condition [27];
  - *W_dry_* is the weight of the solid specimen, in dry condition;
  - *L* is the length of the solid specimen, equal to 75 cm.
2. Density
  (4) $$V_{dry}=\frac{W\left(a\right)}{\rho }-\frac{W\left(a\right)-W_{dry}}{\rho \left(fl\right)}$$
  (5) $$\rho_{dry}=\frac{W_{dry}}{V_{dry}}$$
  where:
  - *V_dry_* is the volume of the specimen in dry condition, in cm^3^;
  - *ρ_dry_* is the density of the specimen in dry condition, in g/cm^3^.
3. Cross section and diameter of the yarn
  (6) $$A_{yarn}=\frac{TEX}{\rho_{dry}}$$
  (7) $$D_{dry}=\sqrt{\frac{4\times A_{yarn}}{\pi }}$$
  where:
  - *A_yarn_* is the area of the yarn, in mm^2^;
  - *D_dry_* is the equivalent diameter assessed assuming a circular cross section of the specimen in dry condition, in mm.
4. Water Absorption ratio
  (8) $$Abs=\frac{W\left(a\right)-W_{dry}}{W_{dry}}\times 100$$

### 2.3. Mechanical Characterisation of Natural Yarns

**Figure 2:**
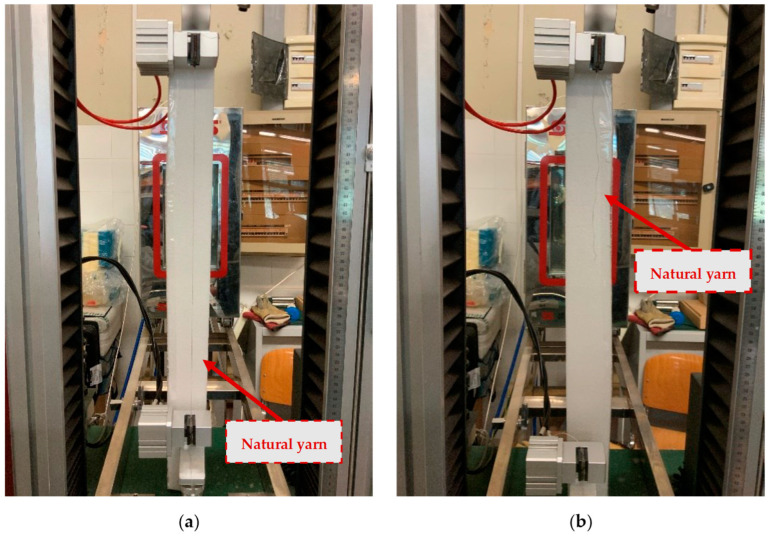
Mechanical characterisation of natural yarns (Flax): Before (**a**) and After (**b**) testing.

The tests were carried out with the aim of obtaining a mechanical characterisation of the yarns that complied with the regulation “BS ISO 3341:2000 EUR [30], which refers to machines with a constant rate of elongation (CRE) and flat clamps with an indicated nominal gauge length of 500 mm. Samples were randomly extracted from the textile: 10 yarns were tested for each textile. Tensile tests were performed by means of a CMT4000 SANS Series dynamometer (MTS, Shenzhen, China) in displacement control with a rate of 200 mm/min ± 20 mm/min. (Figure 2). The load and elongation were recorded during the test.

### 2.4. Mechanical Characterisation of Natural Textiles

**Figure 3:**
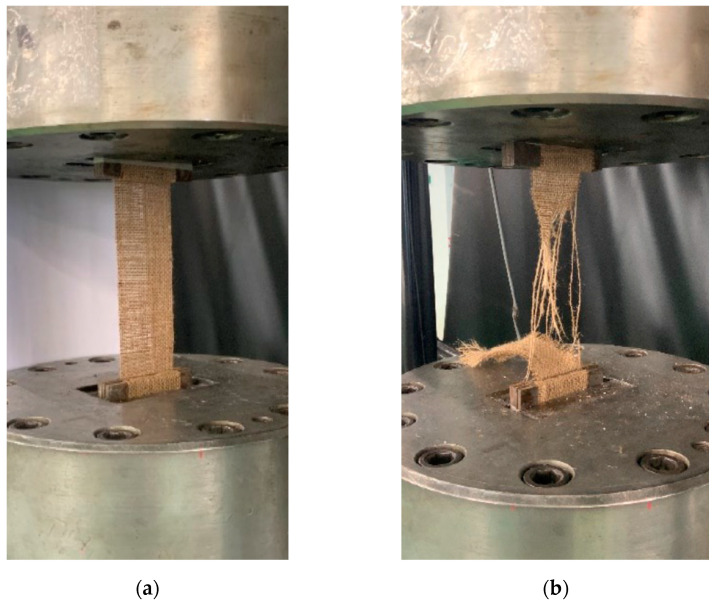
Mechanical characterisation of natural textile (Jute): Before (**a**) and After (**b**) testing.

The tests aimed at obtaining a mechanical characterisation of textiles were run according to the relevant Italian Guideline [10]. Additionally, in this case, the tensile tests were carried out using a displacement-controlled tensile machine (CRE, constant rate of extension testing machine). The dimensions of the specimens were 200 mm in gauge length by 60 mm in width, with 100 mm of anchor length (Figure 3). Given these geometric properties, it is possible to indicate an average number of yarns inside the fabric that are equal, respectively, to 39 for flax samples and 28 for jute samples.

Tensile tests were performed by means of a Zwick Roell Schenck Hydropuls S56, (Silandro, Italy) with a maximum capacity of 630 kN. The load was applied axially to the test piece, with the traverse speed specified by the standard being 0.5 mm/min.

### 2.5. Mechanical Characterisation of Lime-Based Mortars

Flexural and compressive strengths of the lime-based matrix were determined according to a well-known testing procedure defined by EN 1015-11:2019 [31]. The mortar samples were produced in accordance with what is described in the technical sheet [5,6] and then used to produce samples. Three 160 mm × 40 mm × 40 mm prismatic samples were obtained from each batch of mortar during NTRM casting and tested after 28 days of curing for the assessment of the mechanical properties of the hardened matrix.

#### 2.5.1. Flexural Strength

The first performed test was a 3-point bending test where the support configuration consisted of 3 cylindrical steel supports, with a length between 45 mm and 50 mm, and 10 mm in diameter. The two places at the base of the apparatus, on which to place the specimens, were spaced 100 mm apart (with an error of 0.5 mm). The third element, the same as the previous ones, was placed centrally at the top and acted as the support used to apply the load on the mortar sample [31].

The sample was subjected to the loading rate in the range between 10 N/s and 50 N/s, up to the breaking point. The value recorded during this test coincides with the maximum applied load in N. So, the flexural strength, *f*, in N/mm^2^, is [31]:

(9) $$f=1.5\times \frac{F\times l}{b\times d^{2}}$$

where:

- *f* is the flexural strength;
- *F* is the maximum load applied to the specimen, in N;
- *l* is the distance between the axes of the support rollers, in mm;
- *b* is the width of the specimen, in mm;
- *d* is the depth of the specimen, in mm.

#### 2.5.2. Compressive Strength

As reported in paragraph 9.1.2 of the EN 1015-11:2019 (E) standard [31], “the plates shall be 40.0 mm ± 0.1 mm long × 40.0 mm ± 0.1 mm wide and 10.0 mm ± 0.1 mm thick. The dimensional tolerance for the width shall be based on the average of four symmetrically placed measurements. The flatness tolerance for the contact faces shall be 0.01 mm.”

The maximum applied load was then recorded, in N, in order to calculate the compressive strength: this is obtained by dividing this value by the section area of the specimen, nominally 1600 mm^2^.

### 2.6. Mechanical Characterisation of Natural TRM Systems

The reference standards for these tests are two. First, the Guideline for the Qualification of FRCM was still considered; in addition, a document drawn up by a RILEM Technical Commission, called “Recommendation of RILEM TC 232-TDT: test methods and design of textile reinforced concrete” [13], was referred to for the creation of the specimens.

The first operation coincides with the preparation of two types of composites:

- *Limepor MT* matrix with jute fabric reinforcement;
- *Limepor EDO* matrix with flax fabric reinforcement.

Referring to the geometry of the samples, these have a rectangular shape, with a length of 500 mm and a width of 60 mm. The thickness of the specimens must not exceed 6 mm. The direction of the textile yarns was parallel to the axis of the test piece and placed in its middle plane [13].

The procedure performed for the production of the specimens is as follows:

1. Assembly of the formwork necessary for the implementation of the samples;
2. Preparation of the two types of premixed mortar;
3. The laying of a thin layer of release agent, in order to allow the easier removal of the samples, and then of the first layer of mortar;
4. The laying of the fabric band;
5. The laying of the last layer of mortar;
6. After setting, the specimens were extracted from the formwork.

Once 28 days had elapsed, and the mortar had properly hardened, it was possible to pass the actual test. The system adopted in this study was inspired by the RILEM document [13].

An anchoring system was also provided consisting of two steel plates for each end, fixed to the TRM specimen with resin, in order to guarantee the effective seal of the anchor. The resin used was *Kimitech EP-IN* [32]. The plates are anchored to the testing machine (Zwick Roell Schenck Hydropuls S56, Silandro, Italy) by means of a hinge system specifically designed to allow the out-of-plane rotations and, hence, prevent bending and twisting moments from being transferred to the specimen in tension.

Turning to the characteristics of the test, the load must be applied with displacement control mode. In this regard, the speed of the crosshead was set equal to 0.2 mm/min. During the test, the load and displacement values of the mobile crosshead were recorded. The data concerning the thickness of the specimen brought to break were also recorded, in 3 distinct points (the center and the two ends), in order to obtain an average value.

**Figure 4:**
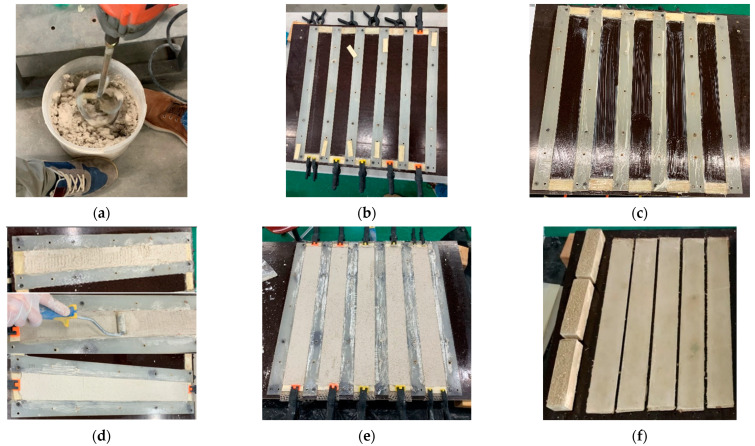
Preparation of TRMs specimens: (**a**) Making of mortars; (**b**) Formworks; (**c**) Laying of release agent; (**d**) Making of NTRM; (**e**) NTRMs specimens before hardening; (**f**) End of NTRMs making process.

The first layer of mortar (Figure 4a), of about 2.5 mm, was applied on the base of the mold, then the flax strip was placed on it, ensuring that it was clamped along two edges of the specimens, to be kept as stretched as possible. The textile was pressed by means of a roller to guarantee the full impregnation of the fabric within the mortar (Figure 4d). The next layer of mortar, with the same thickness as the first one, was placed on it. The application of the outer layer of the mortar was performed by thoroughly smoothing it (Figure 4e,f).

## 3. Results

The present section summarizes the main results obtained in this study. It is worth mentioning that, in order to present a clear distinction between the flax and the jute textile, the “red” representations are always associated with the Jute fabric; meanwhile, the “blue” ones refer to the Flax-based samples.

### 3.1. Physical Properties of Natural Yarns

#### 3.1.1. Linear Density and Density

**Figure 5:**
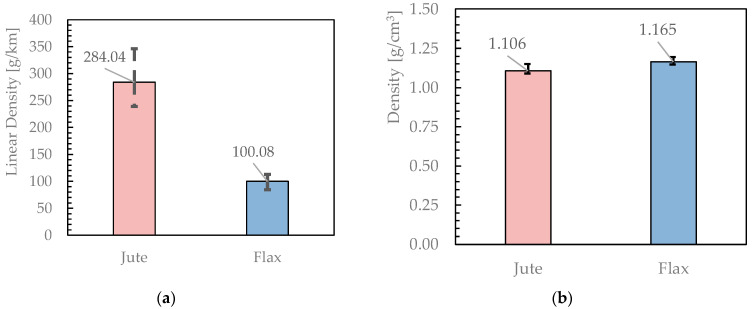
Linear density (**a**) and Density (**b**) of jute and flax yarns.

Starting from the data obtained from the measurements relating to the weight of each yarn, it was possible to determine the linear density values for each of the 10 tested samples according to Equation (3). A similar procedure was carried out with regard to the data obtained from the test that used the hydrostatic thrust to determine the density of the fabric according to Equation (4). Figure 5 reports the related results expressed in TEX and g/cm^3^, respectively. It shows that Jute fibres have a higher linear density than Flax fibres, with an almost similar density. This points out that the Jute fibres are generally wider in diameter than Flax ones. Moreover, the wider error bars in Figure 5a point out a more marked variability in the diameter of Jute fibres.

**Figure 6:**
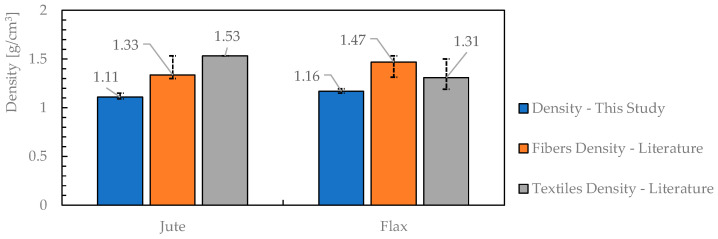
Comparison between density mean values.

Moreover, it is interesting to compare the values obtained for the density of the two types of fabric with the literature values [1,33,34,35,36,37,38]. The comparison is reported in Figure 6. The lower value registered in this study can be associated with the different test methods for measuring the density of the natural yarns. In fact, as also reported in the previous section, the density was measured by using distilled water while, in some cases in the literature, soybean oil is employed.

#### 3.1.2. Other properties: Cross Section and Absorption

**Figure 7:**
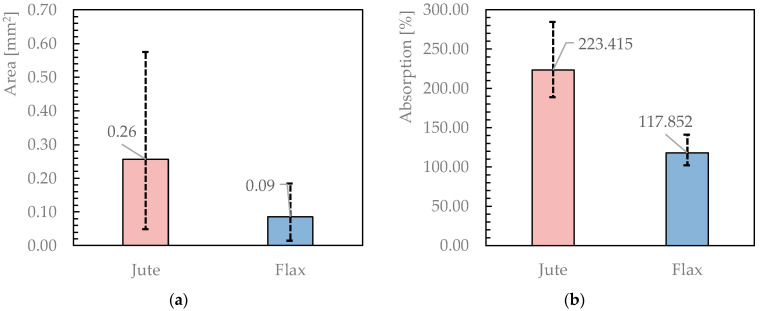
Jute and flax ideal Cross-section (**a**) and Absorption (**b**).

Based on the calculation of density and linear density, it was possible to evaluate the ideal cross-section of the yarn. The results obtained herein are shown below (Figure 7), with the mean value and error bars, the latter highlighting the significant variability of the geometric properties of Jute fibres. Moreover, the degree of water absorption was also derived according to Equation (9).

As regards the maximum and minimum standard deviation reported in the two cases studied for the cross-section, these certainly have a much higher variability than in the properties previously reported, which is probably due to the nature of the calculation carried out; in fact, in this case, the reference is to an ideal area, which, therefore, considers the yarn as a perfect cylinder with all the ensuing consequences. Furthermore, the error found for the jute is greater than the error obtained for the flax yarn: this aspect fits perfectly with the description made of the two types of fabric (the more irregular and “frayed” the jute, the more regular and compact the flax).

### 3.2. Mechanical Properties of Natural Yarns

#### 3.2.1. Tensile Strength

**Figure 8:**
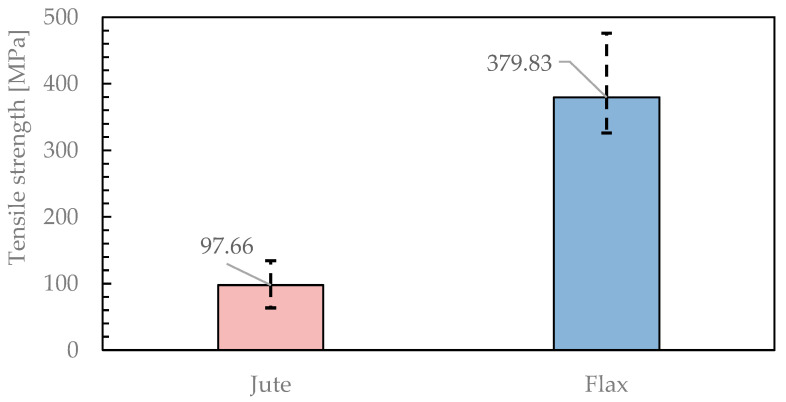
Maximum average tension recorded for flax and jute yarns.

Regarding the mechanical properties of the yarns, Figure 8 represents the results of the tensile tests carried out on 10 samples of Jute and Flax fibres. Specifically, the experimental results are plotted in terms of both average values and error bars: the latter highlighting the minimum and maximum strengths obtained from the tests.

Therefore, the Flax fibres are almost four times stronger in tension than the Jute ones. This can also be regarded as a result of the highest homogeneity of the material, which derives from the lower cross-section area and the reduced variability of its values, as already pointed out in Figure 7.

**Figure 9:**
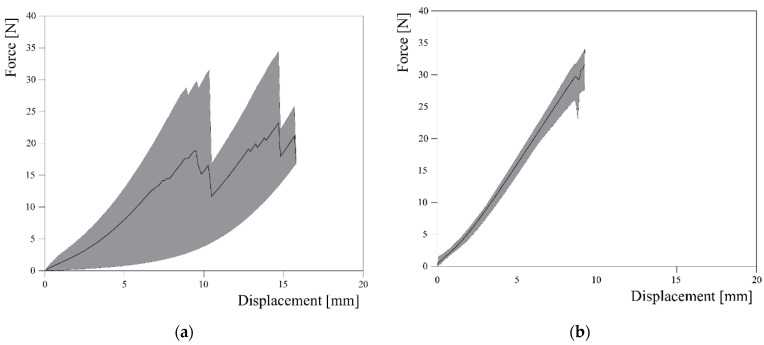
Envelope area between maxima and minima for the Jute yarn (**a**) and Flax yarn (**b**).

An interesting graph that it was possible to extrapolate from the available data obtained from Jute curves is shown in Figure 9, where the shaded areas represent the envelope of the force-displacement curves obtained from experimental tests and the black lines correspond to their average in terms of forces. Once again, the highest variability of the tensile response of Jute fibres is clearly highlighted.

#### 3.2.2. Elastic Modulus

**Figure 10:**
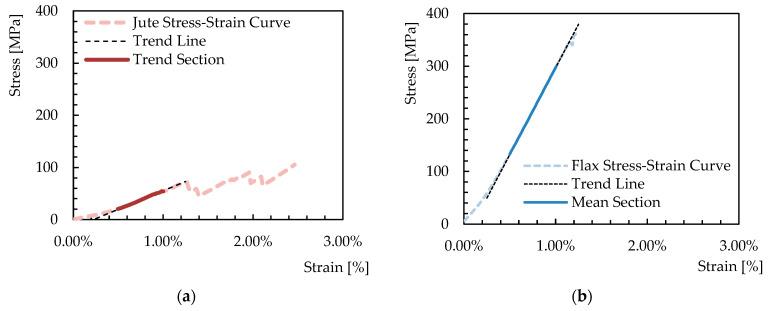
Stress–strain curves and linear branch from which Young’s modulus was determined for Jute (**a**) and Flax (**b**) fabrics.

**Figure 11:**
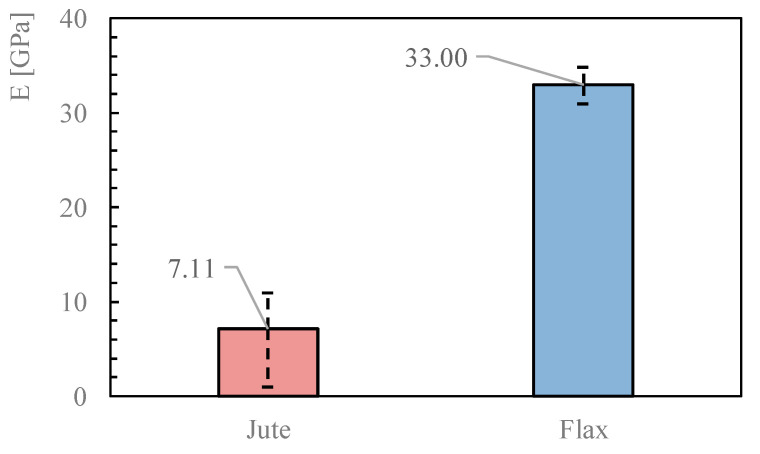
Mean value for Young’s modulus.

The stress–strain curves can be easily drawn by elaborating the force-displacement curves plotted in Figure 9. In fact, the graphs in Figure 10 represent the obtained curves and highlight the almost linear behavior observed between 0.5% and 1.0% of the axial strain, which is considered a determination of Young’s modulus of the fabric, whose values are also represented in Figure 11 in terms of the average values and error bars for both the Jute and the Flax fabrics.

**Figure 12:**
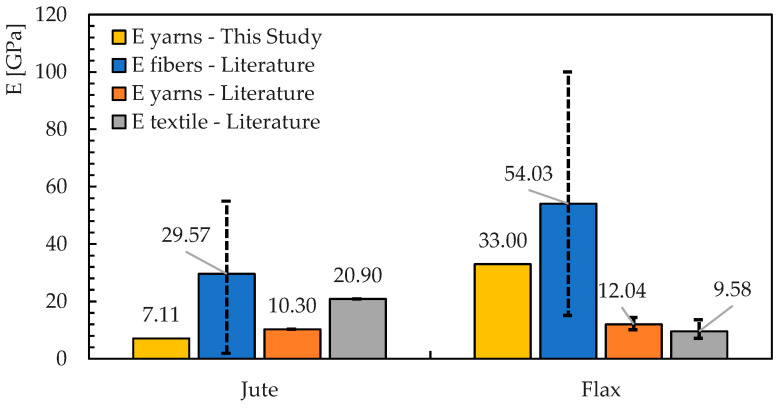
Trendline from which Young’s modulus has been extrapolated for flax fabric.

Additionally, in this case, it is possible to compare these values with what is reported in the literature (Figure 12) [1,33,34,35,36,37,38].

The values obtained from the Jute yarns are similar to other results available in the literature. Conversely, Young’s modulus obtained for the Flax yarns is significantly higher. This comparison, on the one hand, confirms the importance of carrying out a deep geometric and mechanical characterisation for natural fibres and fabrics. On the other hand, it provides readers with some of the fundamental information needed to understand the behavior that will be described in Section 3.4 for the NTRM under consideration.

### 3.3. Mechanical Properties of Natural Textile

**Figure 13:**
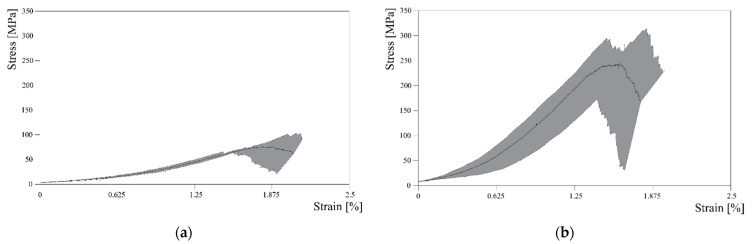
Envelope area between maxima and minima for Jute (**a**) and Flax (**b**) fabric.

The mechanical characterization of the fabric led to the average stress–strain curves and related envelop plotted in Figure 13.

As can be seen, the recorded results do not show high variability, at least up to a deformation value of approximately 1.5%. After this value, however, the discrepancy between the average value and the maximum and minimum that has been recorded increases significantly: this can be linked to what happens during the test itself. In fact, if the sample is observed during the test, it is possible to see that the individual yarns making up the fabric band gradually break without a precise pattern, which could justify this variability.

Unlike what was observed for the jute fabric, the band related to the flax fabric is affected by a more pronounced variability, which appears to increase as the recorded deformations increase.

**Figure 14:**
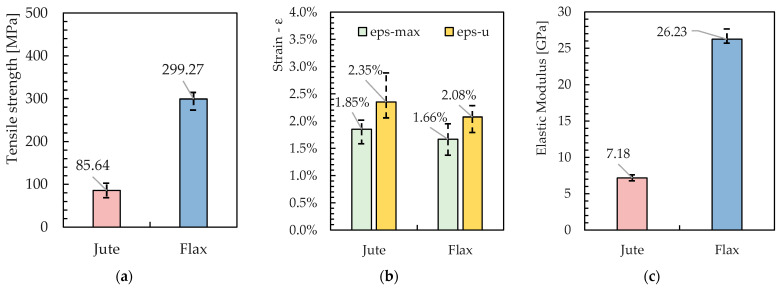
(**a**) Average of the maximum tensile strength, for jute and flax; (**b**) Average of the maximum Young Modulus, for jute and flax; (**c**) Maximum strain and ultimate strain at break for jute and flax.

In addition, a summary of the results obtained following the tests carried out on the five strips of jute fabric is shown in the following tables. In particular, a reference is made to the maximum tension values reached, with the relative deformation connected to it, and also the value of the ultimate deformations reached by the fabric band (Figure 14). These values are indicated, respectively, with:

- σ_max_: Maximum tension reached during the test;
- ε_σmax_: Deformation reached when maximum tension occurs;
- ε_u_: Ultimate deformation reached before failure.

Similar to the yarns, and also in this case, there is a difference between the two tensions in the favor of flax. As for the Young Modulus, it is confirmed that the jute fabric was much softer (almost four times) than the flax fabric. Therefore, the Jute fabric is significantly less rigid in tension than the Flax fabric, which, as will be demonstrated in Section 3.4, plays a significant role in the structural behavior of the NTRM composite system in tension.

### 3.4. Mechanical Properties of Lime-Based Mortar

**Figure 15:**
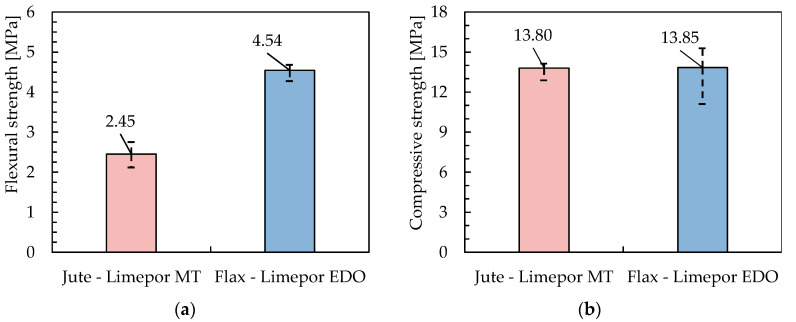
Flexural strength (**a**) and Compressive strength of mortars (**b**).

Figure 15 shows the results of flexural and compressive tests carried out according to the procedure described in Section 2.5.

As can be seen, the two mortars have similar behavior in compression, but the *Limepor MT* mortar (which is meant to be reinforced by a Jute fabric) has a significantly lower flexural strength than the *Limepor EDO* mortar (which is coupled to Flax fabric). This can be explained by the fact that EDO mortar is characterised by a smaller value of the maximum aggregate diameter (0.6 mm) with respect to the MT one (3 mm). However, in principle, a low tensile strength of MT can be appropriate in its coupling with Jute fabrics, which are also characterised by low axial stiffness and strength.

### 3.5. Mechanical Properties of NTRM

This section presents the results obtained following the procedure presented in Section 2.5. In particular, the results presented in graphical form represent the stress–strain curves obtained for the NTRM specimens, respectively, when reinforced with jute and flax fabric. The stress was evaluated by considering the whole cross-section of the resulting NTRM composite. As regards the geometric characteristics of the tested specimens, these have a defined gauge length equal to 200–300 mm. Table 2 and Table 3 summarise the geometric characteristics of the NTRM specimens, made, respectively, of *Limepor MT* and jute fabric and of *Limepor EDO* and flax fabric.

**Figure 16:**
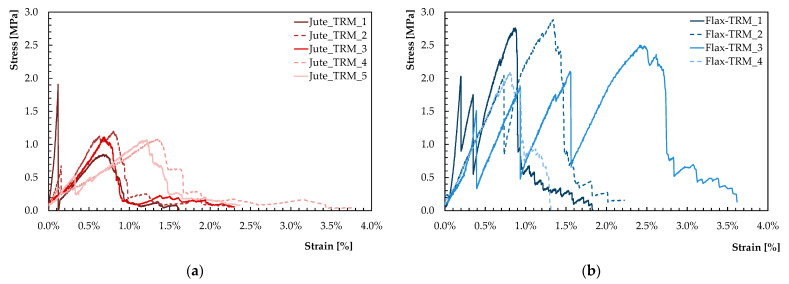
Stress–strain curves of Jute-TRM (**a**) and Flax-TRM (**b**).

Figure 16 depicts the stress–strain curves obtained from the tensile tests on the TRM specimens.

By observing the different curves obtained and comparing them with other reference studies [14,23,26], it is possible to identify, to different degrees, some common aspects. Specifically, as expected, three main stages can be observed [19].

The first stage corresponds to an initial elastic phase of the specimen and ends in the correspondence of the first peak and drop. The entity of this peak mainly depends on the type of mortar used. In fact, Figure 16 shows that the peak is generally higher for the Flax-TRM than for the Jute-TRM, which is consistent with the higher flexural strength exhibited by the respective mortars (Figure 15a).

The second stage is represented by a sequence of raising branches and abrupt drops, the latter corresponding to the opening of a newly formed crack, which happens as the strength reaches values close to the first stage peak. Figure 16 shows that this stage is more clearly developed in the case of Flax-TRM, whereas it is almost absent for Jute-TRM. This means that the Flax-TRM is characterised by a more pronounced interaction between the matrix mortar and the reinforcing fabric, which is the essential feature of a composite system. In other words, from an engineering standpoint, the Flax-TRM more clearly behaves as a composite system than the Jute-TRM under consideration in this study.

The third stage arises when all the cracks are formed on the TRM system the total number of them (or equivalently, their average distance) is affected by both the adhesion between fabric and matrix and the tensile strength of the latter. As the cracks are stabilised, the tensile strength of the TRM is controlled by the reinforcing fabrics, which is why in Figure 16, for both Jute and Flax, the final branch of the stress–strain curve has a fairly linear shape characterised by similar slopes and ultimate strengths. In this regard, Figure 16 confirms that the Flax fabric is stiffer and stronger than the Jute fabric considered in this study.

**Figure 17:**
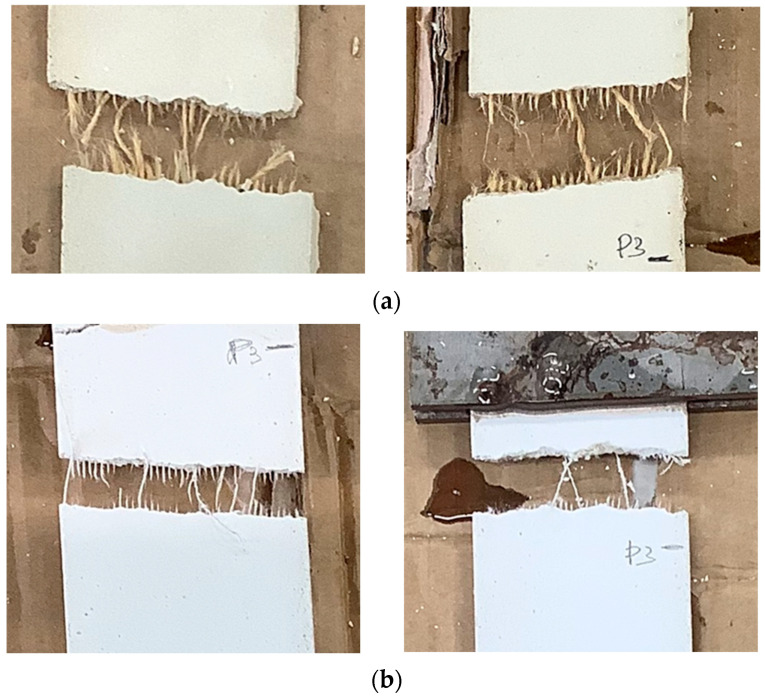
Example of Jute-TRM (**a**) and Flax-TRM (**b**) break.

Finally, after the third stage, the curves reach zero. This coincides with the breaking of the element in one of the cracks that opens during the loading phase. As can be seen from Figure 17, the breakage of the latter occurred following the breakage of the internal reinforcing fabric.

#### Comparisons in Terms of TRM Strength

**Figure 18:**
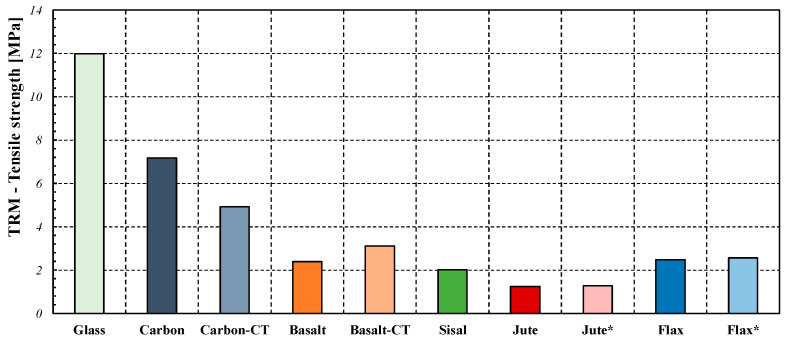
Comparison between different FRCM tensile strength. * Present Study.

It is also relevant to compare the results obtained in the present study with what is reported in the literature. To this aim, Figure 18 shows a bar chart reporting the average values of the ultimate strengths obtained from tensile tests on TRM systems with hydraulic lime-based matrices and one single layer of reinforcing fabric. Specifically, the cases of both more conventional fibres (i.e., glass, carbon, or basalt) and natural materials (i.e., sisal, flax, and jute) are taken into account [2,14,33,37,39,40,41]. All the results included in the assembled database are obtained from tensile tests executed in compliance with the RILEM recommendation [13] and adopting both the “clevis” system and a free length ranging from 300 mm to 370 mm.

The abbreviation “CT” reported in some of the labels of the bar chart in Figure 18 (i.e., for carbon and basalt) corresponds to “coated”: it indicates the cases in which the reinforced nets have been covered with a layer of epoxy resin. As can be seen, for both Jute and Flax fabrics, the resistance values obtained in the present study are very close to the average ones reported in the literature. As far as the comparison with more conventional materials is concerned, it is possible to note that carbon and glass fibers, in any case, express much higher strengths.

## 4. Conclusions

This paper reported the results of an experimental study, which intended to characterise the geometric properties and the mechanical behavior of TRM systems realised with sustainable constituents such as hydraulic-lime mortar and natural fibres. Specifically, the present study aimed at determining the engineering properties of two TRM systems (realised by coupling different mortars and fabrics) with the aim of providing readers with empirical evidence of the influence between the geometric and physical properties of the constituents and the resulting mechanical behavior of the Jute- and Flax-TRM systems under consideration.

The main quantitative findings obtained from the experimental tests can be summarised as follows:

- The Jute fibre yarns have a transverse section bigger than the Flax yarns; specifically, the area of the former is almost three times bigger than the latter, yet it is affected by significant variability;
- The bigger section area also leads to both higher (almost double) absorption capacity and (almost three times) lower tensile strength of the Jute fibres with respect to the Flax fibres;
- Moreover, flax yarns are significantly stiffer in tension than jute fibres, as the Young modulus ratio is in the order of five between them;
- The tensile response of NTRM is greatly affected by the aforementioned properties of the yarns and textile, as the tested Flax-TRM tends to outperform Jute-TRM in terms of both maximum bending strength and overall post-cracking toughness.

Further quantitative results are reported throughout Section 3, which are addressed to the reader for the sake of completeness. However, besides the specific single results, the experimental tests carried out on the Flax-TRM show that it actually behaves as a composite system, as the three stages expected in the tensile response are reasonably well apparent. Conversely, the tested Jute-TRM system needs to be improved in terms of stiffness and strength balance between the mortar matrix and the internal reinforcement: including two layers of Jute fabrics may be a first move to try and obtain a behavior more distinctly to that of a two-phase composite system.

However, further studies are needed to better understand how the mechanical response of the NTRM systems relates to the geometric and mechanical properties of its constituents and how the former can be affected by the deterioration of the latter, possibly induced by exposure to aggressive environmental conditions and observed by the means of microstructural (SEM and EDX) analyses.

## Figures and Tables

**Table 1:** Geometric characteristics of the flax and jute textile.

	X Direction	Y Direction
Sample Dimension	50 mm	50 mm
Jute	28 yarns	28 yarns
Flax	32 yarns	31 yarns

**Table 2:** Geometric characteristics of Jute-TRM.

Characteristic	Jute-TRM_1	Jute-TRM_2	Jute-TRM_3	Jute-TRM_4	Jute-TRM_5
Thickness [mm]	6.50	6.33	6.83	6.33	6.33
Length [mm]	300	300	300	200	200

**Table 3:** Geometric characteristics of Flax-TRM.

Characteristic	Flax-TRM_1	Flax-TRM_2	Flax-TRM_3	Flax-TRM_4
Thickness [mm]	5.33	5.00	5.17	5.00
Length [mm]	300	300	200	300

## Data Availability

Not applicable.
